# Theranostic Mesoporous Silica Nanoparticles Loaded With a Curcumin-Naphthoquinone Conjugate for Potential Cancer Intervention

**DOI:** 10.3389/fmolb.2021.670792

**Published:** 2021-05-20

**Authors:** Lara G. Freidus, Pradeep Kumar, Thashree Marimuthu, Priyamvada Pradeep, Yahya E. Choonara

**Affiliations:** Wits Advanced Drug Delivery Platform Research Unit, Department of Pharmacy and Pharmacology, School of Therapeutic Sciences, Faculty of Health Sciences, University of the Witwatersrand, Johannesburg, South Africa

**Keywords:** curcumin, naphthoquinone, cancer, mesoporous silica nanoparticles, theranostics, fluorescence

## Abstract

A novel theranostic molecule, derived from curcumin (Cur) and naphthoquinone (NQ), allowing for cancer targeting, detection and treatment was previously described and termed CurNQ. To allow for enhanced theranostic capabilities, advanced drug delivery techniques are required. To this end, mesoporous silica nanoparticles (MSN) were synthesized and CurNQ was loaded into its pores to form the novel nanosystem MSN_CurNQ. The formation of the nanosystem aimed to augment the drug delivery of CurNQ through the EPR effect and sustained release. Moreover, the loading of CurNQ into its pores, formed a fluorescent nanoparticle that can be tracked, detected and visualized. Herein, the synthesis of a novel nanosystem is described and its theranostic potential are explored *in vitro*. MSN with an average size of 108 d.nm, a zeta potential of −42 mV and a PDI of 0.150 were synthesized and were impregnated with CurNQ to form the novel nanosystem MSN_CurNQ. MSN_CurNQ was demonstrated to have pH-responsivity whereby after 96 h, at pH 7.4, 31.5% of CurNQ was released from the MSN compared to 57% release at pH 6.8, corresponding to an increase of 25.5% in release with a 0.6 pH drop. The innate fluorescence was then characterized through confocal and fluorescence microscopy. Microscopy images illustrated the distinct, high intensity innate fluorescence with a high background to target ratio, thus confirming detection capabilities and potentially extending MSN_CurNQ’s application to molecular imaging purposes. Moreover, the chemotherapeutic potential of MSN_CurNQ was demonstrated as cell viability was reduced to below 50% in OVCAR-5, CACO-2, CHLA, and MCF-7 cell lines. Furthermore, MSN_CurNQ displayed tumor specific toxicity whereby the cell viability was reduced to a far greater extent in the cancer cell lines compared to a healthy fibroblast cell line (*p* = 0.000). Indeed, the novel MSN_CurNQ nanosystem has potential for applications in cancer targeting, detection and treatment.

## Introduction

Cancer is set to become the leading cause of death globally and is the biggest impediment to increased life expectancy in the 21st century as the global incidences and mortality rates are forever increasing (Bray et al., 2018). Even with the multitude of research and resources dedicated to cancer, the cancer burden has not eased (Maruthappu et al., 2017). Clearly, new and improved strategies for cancer diagnostics and targeted treatment is an immediate necessity.

The search for new chemical entities for cancer treatment is ongoing. New cancer therapies should circumvent the issues that plague current chemotherapeutics, some of which include, high toxicity, non-specificity, cancer resistance and late diagnosis (Tran et al., 2017; Haider et al., 2020). One of the current trends in cancer research is the development of advanced platforms using nanotechnology that allows for simultaneous diagnostics and treatment, this field of research is referred to as theranostics (Saroj and Rajput, 2018). Concurrent diagnostics and treatment would allow for earlier detection and treatment of malignancy, resulting in improved prognosis and lower mortality rates (Wicki et al., 2015).

Theranostic intervention is often achieved using nanotechnology. Nanomedicine has become of great interest in the field of cancer research on account of enhancements to drug delivery mechanisms (Shi et al., 2017). A significant barrier to cancer treatment is the non-specificity of treatment which induces severe side effects and reduces treatment efficacy (Hu et al., 2016; Haider et al., 2020). The targeting of treatment to cancer cells would reduce systemic toxicity and increase treatment effectiveness and thus is of immense interest (Wicki et al., 2015). The tunable and controllable drug delivery that can be achieved using nanomedicine has propelled this field forward into the spotlight of cancer intervention (Youn and Bae, 2018).

Leaky tumor vasculature is hallmark feature of cancer. This induces nanoparticles to leak out of tumor blood vessels (Shi et al., 2017). This phenomenon does not occur in healthy vessels owing to well-organized branching and regularly spaced capillary beds. This highly structured vessel is not apparent in tumor vasculature on account of rapid tumor growth, allowing for the leaking of nanoparticles into the tumor interstitium. Concurrently, poor tumor lymphatic drainage enables nanoparticle accumulation and enhanced retention (Baeza et al., 2016; Tran et al., 2017). The automatic accumulation of nanoparticles in tumor tissue has been well documented and is referred to as the Enhanced Permeation and Retention (EPR) effect (Shi et al., 2017). This effect has been the rationale behind the field of nanomedicine for cancer intervention. EPR allows for the passive targeting of nanoparticles to tumor tissue (Ivey et al., 2016). The addition of an active targeting strategy in conjunction with EPR can allow for enhanced tumor targeting capabilities.

Mesoporous silica nanoparticles (MSN) are a class of nanoparticles that have been widely studied for drug delivery applications. Silica is one of the most abundant natural resources and has been deemed safe and is widely used in food additives, pharmaceutical products and cosmetics (Bernardos and Kouřimská, 2013). With the explosion into nanomedicine research, silica has been the focus of an abundance of research, mainly in terms of drug delivery (Moreira et al., 2016; Watermann and Brieger, 2017). MSN have numerous features that make them ideal for advanced drug delivery, some of which include their solid framework, high surface to volume ratio, are mesoporous for drug loading, their chemically inert nature, along with their biocompatibility, biodegradability and that they show no toxicity *in vitro* (Argyo et al., 2013; Mai and Meng, 2013; Ma’mani et al., 2014; Liu et al., 2016; Alyassin et al., 2020). Moreover, their hydrophilic silanol surface is ideal for the delivery of low solubility drugs and allows for easy surface functionalization (Mai and Meng, 2013). The large pores evenly distributed over the surface of MSN are ideal for drug loading, especially for the loading of hydrophobic drugs. The loading of hydrophobic drugs into MSN has become a standard approach in the delivery of hydrophobic drug molecules. The poor solubility of these drugs is overcome as EPR delivers these loaded drugs directly into the tumor (Ma’mani et al., 2014). These properties of MSN make them ideal for advanced drug delivery applications.

Our previous research demonstrated the development of a novel theranostic molecule for cancer intervention. The novel molecule termed CurNQ, was synthesized through a Williamson ether synthesis reaction between curcumin (Cur) and lawsone (Freidus et al., 2020). Cur and lawsone were selected owing to the plethora of research that has been conducted on their promising anticancer properties, as well as their highly aromatic structures, allowing for fluorescent properties. As per our reported research (Freidus et al., 2020), CurNQ exhibited theranostic properties with potential applications in simultaneous cancer targeting, detection and treatment. CurNQ was demonstrated to have pH specific solubility, whereby at pH 7.4 it remained mostly insoluble, but experienced a large increase in solubility at the pH associated with the tumor microenvironment, pH 6.8. Thus, allowing for cancer targeting through the exploitation of the change in pH associated with malignant transformation (Warburg, 1925). Once soluble, CurNQ displayed intense fluorescence, further allowing for cancer detection. Additionally, CurNQ induced cytotoxicity to two ovarian cancer cell lines, thereby extending its application to cancer therapy. To allow for the use of CurNQ in clinical applications, CurNQ was required to be delivered to the tumor microenvironment and for its fluorescence to be detected. Consequently, the loading of CurNQ into a nanoparticle for enhanced drug delivery is expected to further the theranostic capabilities of CurNQ allowing for enhanced targeting, sustained drug delivery and detection of the fluorescence nanoparticle.

The aim of this research was to further the theranostic capabilities of the previously described novel molecule CurNQ through the synthesis of mesoporous silica nanoparticles and the loading of CurNQ into its pores (MSN_CurNQ). This aimed to form a detectable fluorescent nanoparticle that has additional targeting and therapeutic properties.

## Materials and Methods

### Materials

CTAB—Hexadecyltrimethylammonium bromide (MW−364.45), Pluronic F-127 (MW–12600), TEOS—Tetraethyl orthosilicate (MW–208.33), Phalloidin-Atto 488 (MW- 1472) and DAPI- 4′,6-Diamidine-2′-phenylindole dihydrochloride (MW- 350.25) were purchased from Merck/Sigma Aldrich (Darmstadt, Germany) and were used as received. High purity solvents (> 98%) were purchased and used as received from ACE chemicals (Johannesburg, South Africa). Cell lines were all acquired from Cell Biolabs Inc. (San Diego, United States) and from Fox Chase Cancer Center (Philadelphia, United States). Cell culture consumables, MTT–3- (4,5-dimethylthiazol-2-yl)-2,5-diphenyl tetrazolium bromide, and other cell culture reagents were sourced from Thermo Fischer Scientific (Waltham, United States).

### Mesoporous Silica Nanoparticle Synthesis

**FIGURE 1:**
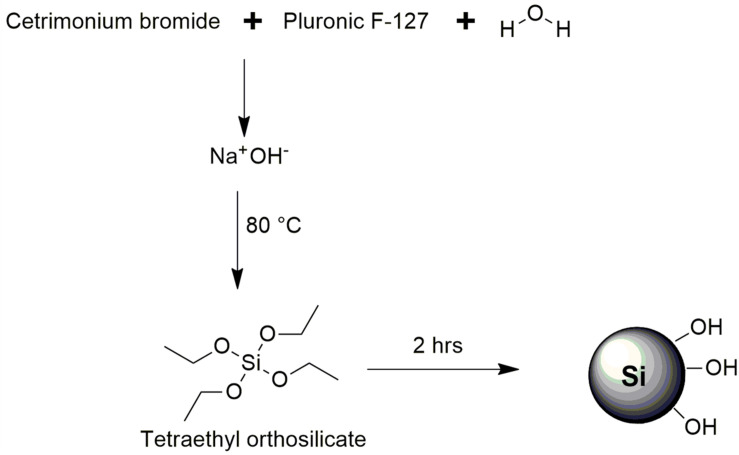
Reaction scheme for the synthesis of MSN.

The synthesis of mesoporous silica nanoparticles (MSN) was performed using a modified method establish by Yang et al. and the synthesis scheme is displayed in Figure 1 (Yang et al., 2006). Equal concentrations of CTAB and surfactant (Pluronic F-127) were dissolved completely in deionized water. 2 M sodium hydroxide was added into the solution and then the temperature of the solution was increased and maintained at 80°C. 6 mL of TEOS was added dropwise at a steady drop rate into the solution and then the solution was stirred rapidly whilst maintaining the temperature at 80°C for 2 h. Subsequently, the solution was immediately filtered and left to dry for 24 h at ambient temperature. The remaining white powder was then collected and calcined at 550°C for 5 h in a furnace to remove the unwanted surfactants.

### Determination of MSN Size and Zeta Potential

Size and zeta potential analyses were performed on the Zetasizer Nano ZS (Malvern, United Kingdom.). Dilute 10 mL suspensions of nanoparticles were sonicated for 30 min at room temperature prior to analysis to reduce particle aggregation. 2 mL of the dispersed samples were filtered into disposable cuvettes and the size, polydispersity index (PDI) and zeta potential values were recorded at 25°C on the ZetaSizer Nano ZS.

### X-Ray Diffraction—Crystallinity Characterization

X-ray diffraction (PXRD) utilizing a variable and fixed slit system was used to determine the crystallinity and atomic spacing and unit cell dimensions of the MSN. This characterization technique was performed on the Rigaku MiniFlex 600 Benchtop X-ray Diffractometer (Rigaku Corporation, Tokyo, Japan). A powdered sample was packed onto the sample holder and was analyzed at a scanning rate of 15° per minute at a diffraction angle range of 3–90° with a degree step of 0.02, a voltage of 40 kV and a current of 15 mA.

### Porosity and Surface Area Determination

Surface area and porosity analysis was performed on MSN using Porositometric Analyzer (Micromeritics ASAP 2020, Norcross, GA, United States). Surface moisture and contaminants were removed through degassing prior to analysis through the insertion of a glass filter rod. Degassing occurred over a period of 9 h and after the degassing process, the MSN were transferred to the analysis port where surface area, pore volume, and pore size data were obtained in accordance with standard BJH and BET gas adsorption method computations (Bawa et al., 2011).

### MSN Morphological Characterization

The FEI Nova Nanolab 600 SEM was utilized for scanning electron microscopy (SEM) which was used to determine nanoparticle morphology and offer particle size confirmation through dynamic light scattering. Powdered sample of the nanoparticles was placed onto an aluminum specimen stub covered with a double-sided carbon adhesive disk and placed under a vacuum, flushed with argon, evacuated and sputter coated with gold for 4 min at 20 kV. SEM images were obtained on a FEI Nova Nanolab 600 FEG-SEM/FIB.

### Loading of CurNQ Into MSN to Create Novel Nanosystem

Mesoporous silica nanoparticles were impregnated with CurNQ and were designated MSN_CurNQ. To achieve this, the methodology previously utilized by Jambhrunkar et al. to load Cur into MSN was followed owing to similarity (Ma’mani et al., 2014). Here 40 mg of CurNQ was dissolved in methanol and mixed with 160 g of MSN particles (1:4 ratio) rapidly for 24 h at room temperature protected from light. The solvent was then slowly removed under reduced pressure using a rotary evaporator, further encouraging CurNQ entrapment. The particles were washed thrice with ethanol and twice with deionizer water to remove unentrapped CurNQ and loaded particles were collected *via* centrifugation at 5,000 rpm for 10 min. The particles were then dried in an oven overnight at 37°C. The above procedure was repeated with Cur to produce MSN_Cur, which was to be used for comparative analyses.

### Determination of Encapsulation Efficiency of CurNQ Within MSN

A mixture containing 40 mg of CurNQ and 160 mg of MSN was added to methanol and was stirred rapidly, allowing for loading to occur (Ma’mani et al., 2014). The UV absorbance of the solution was measured at 429 nm for CurNQ and 425 nm for Cur at varying time points over 48 h. A decrease in absorbance over 48 h was recorded and the concentration of drug uptake was calculated using a standard curve of concentration versus UV absorbance at 429 and 425 nm, respectively (*R*^2^ = 0.99).

Entrapment efficiency values of 54.54 and 32.5% were computed for CurNQ and Cur, respectively. A decrease in UV absorbance indicated successful entrapment owing to CurNQ being impregnated into MSN thereby being removed from the solution.

### Determination of CurNQ Release Profile From MSN at Varied pH

CurNQ release from the MSN nanoparticles was measured at pH 7.4 and 6.8 at 37°C for both MSN_CurNQ and MSN_Cur. One mg/mL of CurNQ-loaded nanoparticles were immersed in a PBS/CTAB solution and the pH of each sample was adjusted to mimic both physiological conditions (pH 7.4) and at the tumor microenvironment (pH 6.8). Samples were incubated and stirred on an orbital shaker incubator at a rate of 50 rpm at 37°C. At 12-h intervals, 1 mL of buffer was removed, and UV absorbance was measured through UV/Vis spectroscopy at 429 nm. Total CurNQ release was then calculated using a standard curve (*R*^2^ = 0.99) for both CurNQ and Cur, respectively. The experiment was performed in triplicate to ensure statistical reliability (*N* = 3). The experiment was concluded after 96-h as no more CurNQ was released after this time.

### *In vitro* Determination of Chemotherapeutic Potential of MSN_CurNQ

Multiple cell lines were used to determine the cytotoxic effects of MSN_CurNQ and MSN_Cur on a variety of cancer types. The following cell lines were cultured namely: OVCAR-5, 3T3, MCF-7, HeLa, CACO-2, HT-29 and CHLA. The cell lines were cultured using standard tissue culture techniques. Cells were either grown in RMPI 1,640 or DMEM, depending on the guidelines specified by the cell line supplier. All media was supplemented with 10% foetal bovine serum (FBS) and 1% Streptomycin and penicillin (Freidus et al., 2020). Cells were incubated at 37°C at 5% CO_2_ atmospheric pressure. Once cells reached 80% confluency, cells were detached and sub-cultured using trypsin (0.05% w/v) and EDTA (0.01% w/v).

### Cytotoxicity Studies Through MTT Assays

Seven cell lines were cultured and the cytotoxicity of MSN_CurNQ and MSN_Cur was evaluated on all seven cell lines, thus allowing for the cytotoxic effect of MSN_CurNQ to be evaluated on several differing cancer types. Cells were cultured as per above conditions. Cell confluency was maintained at 70–80% through standard sub-culturing techniques utilizing trypsin/EDTA harvesting. Thereafter, 1.5 × 10^4^ cells were counted and distributed equally in a 96 well dish and were incubated at 37°C in 5% CO_2_ for 24 h, allowing for cell attachment. Subsequently, cells were treated with varying concentrations of MSN_CurNQ and MSN-Cur. Cells were incubated in treatments for 24, 48, and 72-h time period. After which, 3- (4,5- dimethylthiazol-2-yl)-2,5-diphenyl tetrazolium bromide (MTT) reagent was pipetted into each well and was incubated 4 h. DMSO was then used to solubilize the formazan crystals. All samples were performed in quadruplicate for statistical reliability. Absorbance readings were then recorded on a Perkin Elmer Victor X3 2030 multilabel reader at 570 nm and 690 nm.

### Determination of Fluorescent Properties of MSN_CurNQ Using Confocal Microscopy

The OVCAR-5 cell line was cultured on coverslips until reaching 70% confluency. Cells were then treated with MSN_CurNQ for 24 h, thereby allowing for cellular uptake to occur. After this, Phalloidin-Atto 488 was added, and staining occurred over 30 min. Cells were then washed and fixed through incubation in 5% formaldehyde in PBS for 20 min. After fixation, DAPI stain was added at a concentration of 1 μg/mL and samples were left to incubate for 15 min in the dark. Hereafter, samples were washed thoroughly with PBS to remove unstained DAPI. Coverslips were stored in the dark in PBS. Coverslips were viewed on the Zeiss Laser Scanning Confocal Microscope (LSM) 780 (Oberkochen, Germany). Super resolution microscopy was performed using Airyscan technology as an added on feature to the LSM confocal microscope.

### Cytotoxicity Studies Using High Content Imaging System

Exploiting the use of advanced technological systems such as machine learning, and AI has rapidly advanced the field of cell biology and enables quantitative and qualitative data to be extracted through microscopy images (Wedege et al., 2016). In-depth and detailed data was obtained through rapid microscopy image acquisition and the application of complexed algorithms which were applied using a high content imaging system and provided detailed quantitative and qualitative data on cellular events after treatment with the novel nanosystem. Cells were detached from flasks using EDTA/trypsin and were transferred and distributed equally to a 96-well dish and were then incubated under standard conditions overnight. Cells were then treated with MSN_CurNQ and MSN_Cur at varying concentrations, and cells were left in treatment for 24 h after which, cells were washed with PBS, fixed in 5% formaldehyde for 20 min and then incubated in a 1 μg/mL solution of DAPI for 15 min. Subsequently, cells were thoroughly washed in PBS thus removing residual unbound dye. The 96-well dish was then immediately inserted into the Logos Biosystem CELENA <^®^ X High Content Imaging System which obtained rapid fluorescent microscopy images of each well, and quantitative and qualitative data was obtained from these images through the use of their proprietary software (Geonggi-do, South Korea).

## Results and Discussion

### Synthesis and Characterization of Mesoporous Silica Nanoparticles

Mesoporous silica nanoparticles (MSN) were synthesized and were aimed to act as a delivery platform for CurNQ (Mai and Meng, 2013). Small particles with an average size around 100 d.nm with a substantial negative zeta potential were desired, allowing for the EPR effect as well as for the colloidal stability of the particles. A modified method from Yang et al. (2006) allowed for the synthesis of MSN with the desired characteristics.

**FIGURE 2:**
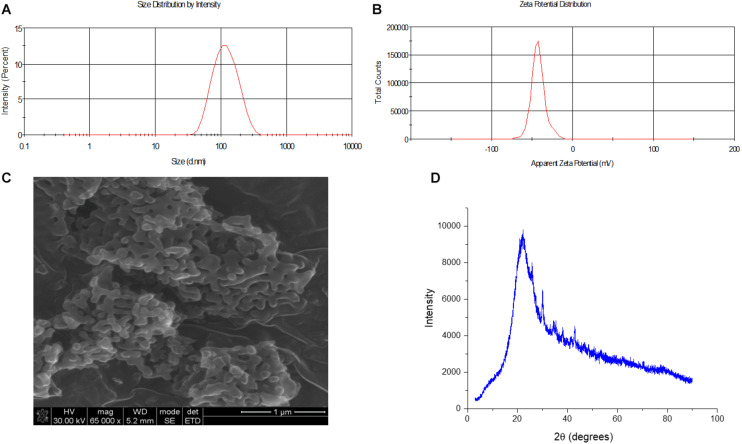
Size, morphology and zeta potential analysis of synthesized MSN. **(A)** Size of MSM determined to be 108 d.nm through dynamic light scattering (*N* = 3) **(B)** Zeta potential of MSN obtained using the ZetaSizer and determined to be –42 mV (*N* = 3) **(C)** SEM microscopy image of the surface of MSN to evaluate surface morphology **(D)** XRD analysis of MSN.

As shown in Figure 2, the average size of the synthesized MSN was determined to be 108 d.nm with a polydispersity index (PDI) of 0.150. Moreover, the zeta potential of the nanoparticles was determined to be −42 mV, confirming the colloidal stability of the nanoparticles with reduced potential for aggregation. Synthesis and particle evaluation were performed in triplicate to confirm reproducibility of desired MSN synthesis. The XRD scan of the synthesized MSN displayed a sharp peak confirming the presence of ordered pores owing to a regular periodic variation of the electron density in the mesoporous silica nanoparticles (Ciesla and Schuth, 1999). The physicochemical properties of the synthesized MSN are summarized in table 1.

**TABLE 1:** Physicochemical properties of synthesized MSN.

Parameter	Size (d.nm)	PDI	Zeta potential (mV)	BET Surface area (m^2^/g)	Pore volume cm^3^/g	Average pore size Å
MSN	108	0.150	−42	30.0883	0.040908	54.3846

The particle size was further confirmed, and the morphology determined through scanning electron microscopy (SEM). This allowed for the surface of the nanosystem to be characterized and to ascertain if the morphology of the nanosystem was uniform. As seen in Figure 2, the particles displayed a uniform morphology, being oval in shape and the average size of the particles observed through the SEM concurred with the zeta analysis results, with an average approximate size of 108 d.nm. (Figure 2). This uniformity was expected owing to the low PDI value which indicated an overall low polydispersity of the sample.

### Impregnation of MSN With CurNQ

Following MSN synthesis and calcination, MSN was impregnated with CurNQ and Cur individually to create the nanosystems MSN_CurNQ and MSN_Cur, respectively. This was achieved through rapid mixing of the MSN and CurNQ in a small volume of methanol protected from light at room temperature for 48 h, allowing for CurNQ to be impregnated in the pores of the MSN. Successful loading of MSN with CurNQ was confirmed through UV/Vis absorbance readings. The absorbance readings were taken before, during and after the 48-h period. A decline in absorbance readings corresponded to the uptake of the drug into the pores of the nanoparticles, thereby leaving the solution and reducing the absorbance of the solution. This uptake was quantified, and the encapsulation efficiency was calculated. Moreover, to further confirm successful CurNQ encapsulation the size of the loaded nanoparticles was measured and compared to the empty nanoparticles. As expected, the size of the CurNQ loaded particles increased upon drug loading from an average of 108 to 214 d.nm. This increase in size was owing to the pores of the particles being impregnated with the product. The above process was repeated for the loading of Cur into MSN, thereby acting as a control. The entrapment efficiencies were calculated to be 54,54 and 32,5% for CurNQ and Cur, respectively.

### Evaluation of pH-Responsiveness of MSN_CurNQ

To support constant cell growth and proliferation, a cancer cell increases glycolysis and bypasses oxidative phosphorylation, this allows for the rapid production of ATP and for cellular proliferation to continue unabated (Warburg, 1925). This phenomenon is known as the “Warburg effect” and is a hallmark feature of oncogenesis. This increase in glycolysis induces the acidification of the ECM, owing to the production of lactate (Warburg, 1925). This results in the slight acidification of the tumor microenvironment and allows for the differentiation between cancerous and healthy tissue (Warburg, 1925). However, this overall pH reduction is slight. Therefore, exploiting this pH reduction to detect and target malignant cells would require a robust detection system which is highly pH sensitive and would elicit a large response to small fluctuations in pH levels. CurNQ displays pH-responsive solubility within the pH change that occurs during malignant transformation and demonstrated a substantial increase in solubility with a shift in pH from 7.4 to 6.8 (Freidus et al., 2020). Moreover, CurNQ was demonstrated to have high fluorescent intensity thereby releasing a strong fluorescent signal once soluble. It was thought that this shift in solubility and concurrent fluorescence could act as a molecular switch allowing for potential tumor targeting and detection applications.

**FIGURE 3:**
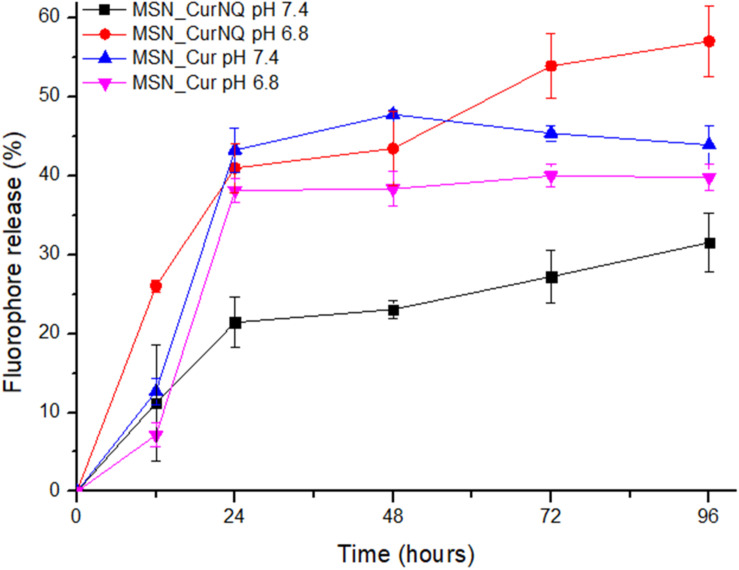
Release assay of CurNQ and Cur from MSN at pH 7.4 and pH 6.8 over a 96-h period.

It was of interest to determine if this molecular switch occurred when CurNQ was loaded onto MSN and if this molecular switch could be exploited for cancer targeting; therefore, a release assay of CurNQ from the MSN was performed at pH 7.4 and pH 6.8. This assay again included MSN_Cur acting as a comparison and allowed for the novelty of this pH switch to be highlighted. As seen in Figure 3, there was a large difference between the release profile of CurNQ at pH 7.4 compared to at pH 6.8, as well as a highly statistically significant different release profile between MSN_CurNQ and MSN_Cur (*p* = 0.0001). The release of CurNQ at pH 6.8 is just less than double its release at pH 7.4. At physiological pH (7.4), there was only 10% release within the first 12 h, with it reaching 20% after 24 h and peaking at 31,5% at 96 h.

This was in stark contrast to the release profile of MSN_CurNQ at pH 6.8. With this small reduction in pH, the novel nanosystem released 43, 45% after 48 h, with the release peaking at 57% at 96 h. This equates to a 25, 5% increase in release occurring in response to a 0.6 reduction in pH. Consequently, the release of CurNQ at pH 6.8 was almost double the release at pH 7.4. This difference in release between the two pH values was highly statistically significant with a corresponding p-value of 0.000. This highly significant *p*-value confirms the effects of pH on the release of CurNQ from the MSN, verifying the pH responsivity and specificity of the nanosystem. Moreover, this highly statistically significant *p*-value emphasizes the robust nature of CurNQ pH-responsivity. Clearly, the change in pH associated with malignant transformation acts as a solubility switch for CurNQ. This pH specificity was not observed for MSN_Cur (*p* = 0.31), and notably MSN_Cur exhibited a slightly greater release at physiological pH, thereby indicating its non-specific pH solubility and its inability to act as a tumor targeting agent.

The release profile aligns with the saturation solubility results presented in our previous research (Freidus et al., 2020). At pH 7.4 CurNQ’s saturation solubility was determined to be 11.5 μM, compared to 20.7 μM at pH 6.8. This drastic increase in solubility in response to small fluctuations in pH levels would allow CurNQ to act as a cancer targeting molecule through a pH specific solubility switch. Figure 3 confirms that this solubility switch occurs once CurNQ was loaded into MSN as the release of CurNQ from the MSN occurred in a pH dependent manner.

The synthesis of CurNQ saw the addition of two quinone moieties and the substantial pH dependent release profile difference between Cur and CurNQ can be attributed to the addition of these quinone moieties. Quinones undergo a reversible two-electron redox reaction and the corresponding redox potential is strongly pH dependent. At acidic pH, reduction occurs and is a single step two-electron two-proton process and this reduction forms a hydroquinone and this hydroquinone form has a greater solubility than the quinone form. Thus, this redox reaction accounts for the switch in solubility and the pH dependent release.

Furthermore, Figure 3 demonstrated the increase of release of CurNQ over a period of 96 h thereby demonstrating sustained release of CurNQ from the MSN. This sustained release is imperative to allow for the fluorescent detection of a tumor and to offer effective treatment upon release, owing to sustained exposure to the anti-cancer agent. Again, this sustained release is not evident in MSN_Cur, as after 48 h, there is no longer further release of Cur, further highlighting the novelty and applicability of the novel nanosystem.

Targeting the slightly acidic tumor microenvironment for tumor targeting, detection and specific drug release has been of great interest for cancer theranostic applications. Several strategies have been investigated by several researchers to achieve this. Such strategies have included the modification of nanoparticles whereby their surface was modified with a pH sensitive compound such as glycol chitosan or through the introduction of protonatable groups. Other avenues include using polymers whereby acid labile bonds are introduced or through an amphiphilic polymer triggered prodrug. Other researchers have used calcium carbonate nanocrystals loaded with cancer drugs that disassociate at the tumor microenvironment, thereby releasing the drug at the desired location (Kanamala et al., 2016; Gulzar et al., 2019). Moreover, many of these strategies utilize the acidic pH of the exosome or lysosome that allows for drug uptake, as the pH in the exosome or lysosome is substantially different to the pH of the cytosol or the microenvironment, thus pH differentiation is easier to achieve compared to the highly sensitive system that is required to detect the change to a slightly acidic microenvironment that accompanies malignancy (Kanamala et al., 2016; Gulzar et al., 2019).

The realization of pH-responsiveness in this study differs to all the strategies mentioned above. To best of our knowledge we are not aware of any active compound that intrinsically offers robust pH sensitivity without the addition of nanomaterials or pH sensitive groups. The simplicity of the pH-responsive solubility of the active compound is unique and is highly novel, especially owing to the sensitivity and alignment to the pH change that occurs during malignancy. Not only is the molecule highly pH specific which is rare, but it offers this specificity at the exact change in pH that is stimulated in response to oncogenesis. Clearly, this is an exceptional finding and can be exploited for tumor targeting and detection applications.

### Evaluating the Innate Fluorescence of MSN_CurNQ and Its Detection Capabilities

The need for novel fluorophores for imaging applications is immense and *in vivo* imaging and detection devices can be applied in numerous and varied applications. The synthesis of a fluorescent compound that is ideal for imaging applications is a large area of research (Freidus et al., 2018). Here, the aim was to extend previous research in the synthesis of a novel fluorescent compound that possesses additional theranostic attributes. The considerations when designing a new fluorescent compound that is ideal for imaging applications is immense. An ideal fluorophore for imaging applications should have an appropriate background to target ratios, high specificity, appropriate absorption and emission wavelengths to avoid tissue autofluorescence, appropriate solubility and fluorescence stability, exhibit cell permeability and have a long fluorescent lifetime (Freidus et al., 2018).

The fluorescent properties of CurNQ were determined through obtaining the absorption and fluorescence spectra, which were measured and recorded on the Shimadzu UV-1800 spectrophotometer and Shimadzu RF-6000 spectrofluorophotometer (Maryland, United States) respectively. The absorption peak was determined to be 429 nm, the excitation peak 595 nm and the emission peak at 670 nm.

CurNQ has a high fluorescence intensity and its pH-responsive solubility would allow it to release a fluorescent signal at the pH of the tumor microenvironment. However, without the loading of CurNQ onto a nanoparticle, the fluorescence of CurNQ cannot properly be traced and visualized, as generalized fluorescence is difficult to pinpoint *in vitro* and *in vivo*. Therefore, the loading of CurNQ onto a delivery vehicle is crucial to exploit its fluorescent capabilities for detection applications. In order to explore these fluorescent capabilities and to determine if MSN_CurNQ can be used for detection and imaging applications, confocal microscopy utilizing super resolution Airyscan technology and fluorescent microscopy on the CELENA^®^ X High Content Imaging System were performed.

These assays were used to verify fluorescence and to determine whether the loaded nanoparticles could be accurately detected and visualized using the innate fluorescent properties of CurNQ; moreover, cellular uptake could be evaluated. Cellular uptake is important as for an active compound to exert its desired effect, it is required to enter the cell. The ovarian cancer cell line OVCAR-5 was utilized for this assay.

**FIGURE 4:**
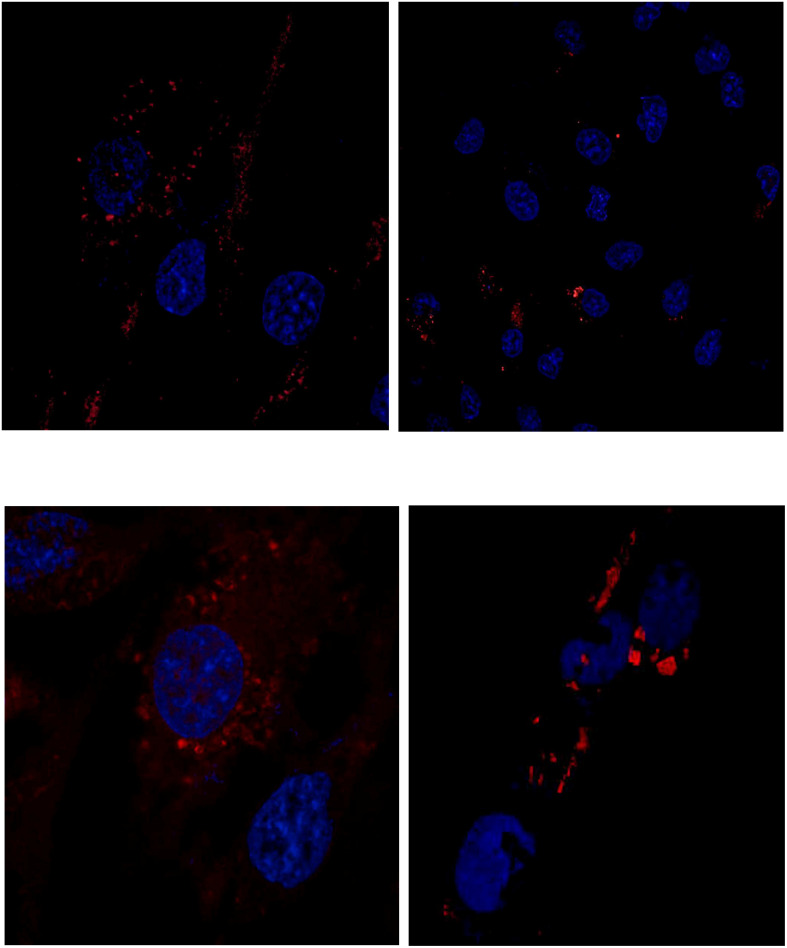
Confocal microscopy images of MSN_CurNQ in OVCAR-5 cells after 24 h. Blue fluorescence is DAPI nuclear staining and red fluorescence is MSN_CurNQ (63× magnification).

**FIGURE 5:**
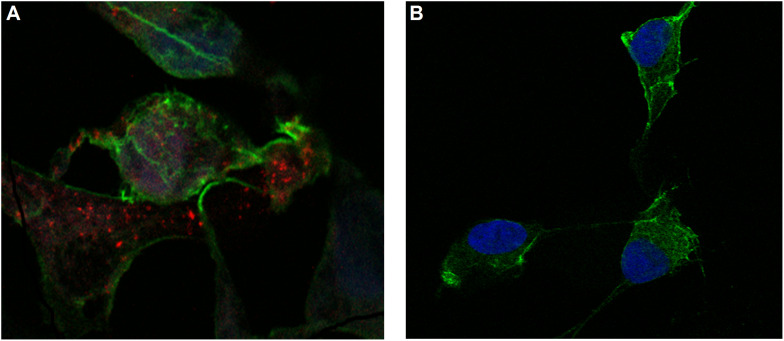
Confocal microscopy images of MSN_CurNQ in OVCAR-5 cells after 24 h (63× magnification). These confocal microscopy images demonstrate that CurNQ is required to be loaded onto a delivery vehicle to allow for fluorescence detection and tracking. **(A)** OVCAR-5 cells treat with MSN_CurNQ for 24 h (63× magnification) **(B)** OVCAR-5 cells treated with CurNQ for 24 h (no MSN). Blue fluorescence is DAPI nuclear staining, green fluorescence is Phalloidin-Atto 488 actin stain and red fluorescence is MSN_CurNQ (63× magnification).

**FIGURE 6:**
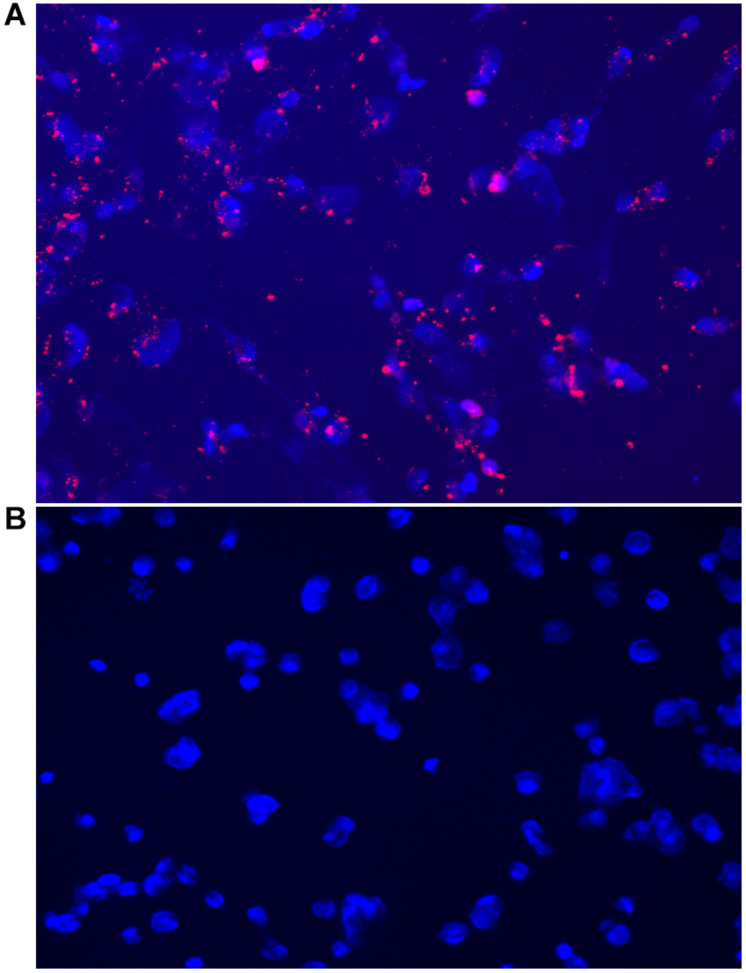
Fluorescent microscopy images acquired on the CELENA^®^ X High Content Imaging System of **(A)** OVCAR-5 cells incubated with MSN_CurNQ for 24 h (20× magnification). Blue fluorescence is DAPI nuclear staining and red fluorescence is MSN_CurNQ. **(B)** Cells given no treatment (control). No red fluorescence is present in the control group, confirming fluorescence is from MSN_CurNQ.

Cells were incubated with MSN_CurNQ for 24 h, to allow for cellular uptake to occur. Subsequently, cells were fixed in formaldehyde and stained with DAPI. The cells were then viewed under the Zeiss LSM 780 confocal microscope (Figures 4, 5) as well as the CELENA^®^ X High Content Imaging System (Figure 6).

As seen in the microscopy images, MSN_CurNQ displayed clear, distinctive, high intensity fluorescence. The fluorescence was distinctive enough to pinpoint small clusters of nanoparticles, with almost no fluorescent blur. Moreover, a high target to background ratio with low background fluorescence and high fluorescence specificity was observe. This is a crucial requirement for a fluorophore effective for *in vivo* imaging. Hence, this distinctive and precise fluorescence with low background poses MSN_CurNQ as a potentially powerful imaging agent. Cell permeability is critical to allow for internal cellular imaging, as well as required for therapeutic applications; as seen in the above images, MSN_CurNQ exhibited cell permeability, and was evenly distributed throughout the cytoplasm. No loss of fluorescent intensity was observed after repetitive imaging over time using the same sample, therefore MSN_CurNQ has a long fluorescent lifetime, has stable fluorescence as was not easily photodegraded or photobleached. These fluorescent properties potentially offer the opportunity to extend the use of MSN_CurNQ for molecular imaging applications.

Moreover, the fluorescent images demonstrate that there was cellular uptake of MSN_CurNQ, but not nuclear uptake. The nanoparticles can be observed to cluster around the nucleus, yet they do not enter the nucleus. Cellular uptake is of great importance, as for a drug to elicit its response, it is required to enter the cell. Clearly, cellular uptake occurs allowing for MSN_CurNQ to elicit its potential anti-cancer effects. Cellular uptake may occur through endocytosis or membrane permeation; however; the method of uptake cannot be determined through the above microscopy images.

As discussed above, it was expected that the inclusion of a nanosystem would be required to realize the potential detection and imaging applications of CurNQ. Therefore, it was of interest to visualize cells treated with the loaded nanosystem MSN_CurNQ and CurNQ treatment alone. As seen in Figure 5, no red fluorescence is observable in cells treated with CurNQ alone, this indicates that CurNQ needs to be loaded onto a nanoplatform for detection capabilities to be realized.

One of the main objectives of this research as to synthesize a molecule with appropriate fluorescent properties to allow for detection and imaging applications. Cur cannot be used for detection or imaging applications owing to its short fluorescent lifetime and its low quantum yield. Furthermore, it undergoes rapid photo bleaching and has a low quantum yield and fluorescent intensity in aqueous solution (Priyadarsini, 2009). Therefore, it was a challenge to improve these fluorescent properties with Cur’s core structure being present in CurNQ and to develop a fluorescent molecule having suitable properties for fluorescence detection applications. Cur has an excitation and emission spectra of 450 nm and 550 nm and these wavelengths would induce tissue autofluorescence if used in *in vivo* applications, therefore a longer excitation wavelength is required to avoid autofluorescence. The synthesis of CurNQ resulted in the excitation and emission wavelengths being significantly red shifted compared to Cur. CurNQ had an excitation and emission wavelength of 595 and 670 nm, respectively, this equates to a red shift of 145 nm. This is noteworthy, as excitation at 595 nm would substantially reduce tissue autofluorescence and would allow for effective *in vivo* imaging. The stokes shift was determined to be 75 nm, this significant gap between the excitation and emission spectra would reduce fluorescence self-absorbance thereby allowing for *in vivo* imaging application (Qi et al., 2018).

The microscopy images demonstrated MSN_CurNQ to have highly precise, distinctive and high intensity fluorescence. Moreover, a high target to background ratio, low fluorescent blur, a long fluorescent lifetime and fluorescent stability indicate MSN_CurNQ to be a potentially powerful diagnostic and imaging agent.

### Evaluating the Chemotherapeutic Properties of MSN_CurNQ Through High Content Imaging

High content imaging is an advanced technique that combines high throughput techniques and fluorescent microscopy to obtain quantitative data from complex biological systems (Zanella et al., 2010). It is a robust method utilizing an automated microscope that allows for rapid, high content image acquisition and analysis (Zock, 2009). Multiple independent measurements can be collected from a single cell or a single cell population. Herein, this advanced technique was employed using the CELENA^®^ X High Content Imaging System. The high content imager was used to measure cell viability in response to MSN_CurNQ treatment; moreover, the number of nanoparticles were counted, enabling the evaluation of the effect of concentration on cell viability.

**FIGURE 7:**
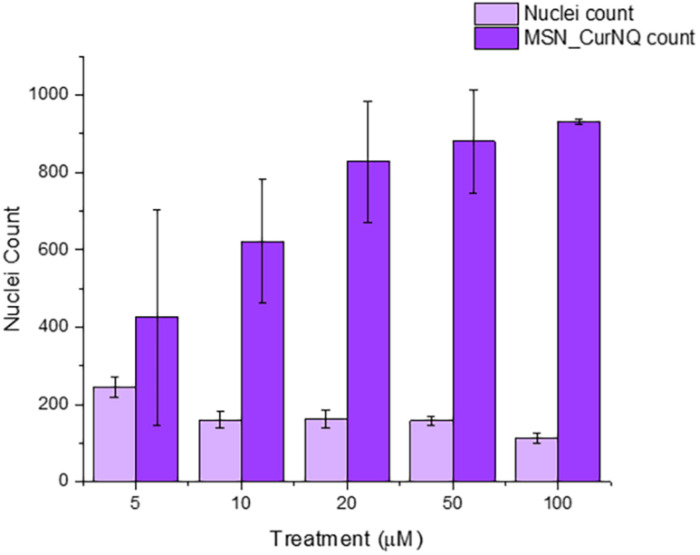
Nuclei count, and MSN count of OVCAR-5 cells treated with varying concentrations of MSN_CurNQ for 24-h. Nuclei and MSN count were quantitatively obtained using a high content imaging system.

As seen in Figure 7, there was a dose dependent response to MSN_CurNQ treatment. With an increasing count of loaded MSN, there was a decrease in cell count, occurring in an inversely proportional manner. Therefore, treatment with MSN_CurNQ resulted in a decrease in cell viability. The visual representation with concurrent qualitative data acquired from the high content imager makes the high content imaging system a powerful tool in evaluating the anti-cancer properties of a particular system and in the drug discovery process. These results suggest the MSN_CurNQ has potential to act as a chemotherapeutic agent.

### *In vitro* Cytotoxicity Assay–MTT

It was of interest to investigate the cytotoxic nature of MSN_CurNQ on a variety of cancer cell types. OVCAR-5 cells were used as the model cell type when investigating the anticancer effects of CurNQ, thus it was necessary that this cell line was carried through to the research on MSN_CurNQ. The inclusion of MCF-7 and HeLa aimed to expand the area of investigation to other female cancer types. Moreover, the additional of varied cancer cell types aimed to give a broad investigational approach to the anticancer effects of MSN_CurNQ on account of the novelty of the nanosystem.

To this end, MTT assays were performed on 6 cancer cell lines and one non-malignant fibroblast cell line. The purpose of these MTT assays were two-fold. Firstly, it was crucial to determine if CurNQ would induce its previously observed potent anti-cancer effects whilst loaded onto the MSN delivery vehicle and secondly to evaluate if the cytotoxicity of CurNQ was translatable to a variety of cancer types.

**FIGURE 8:**
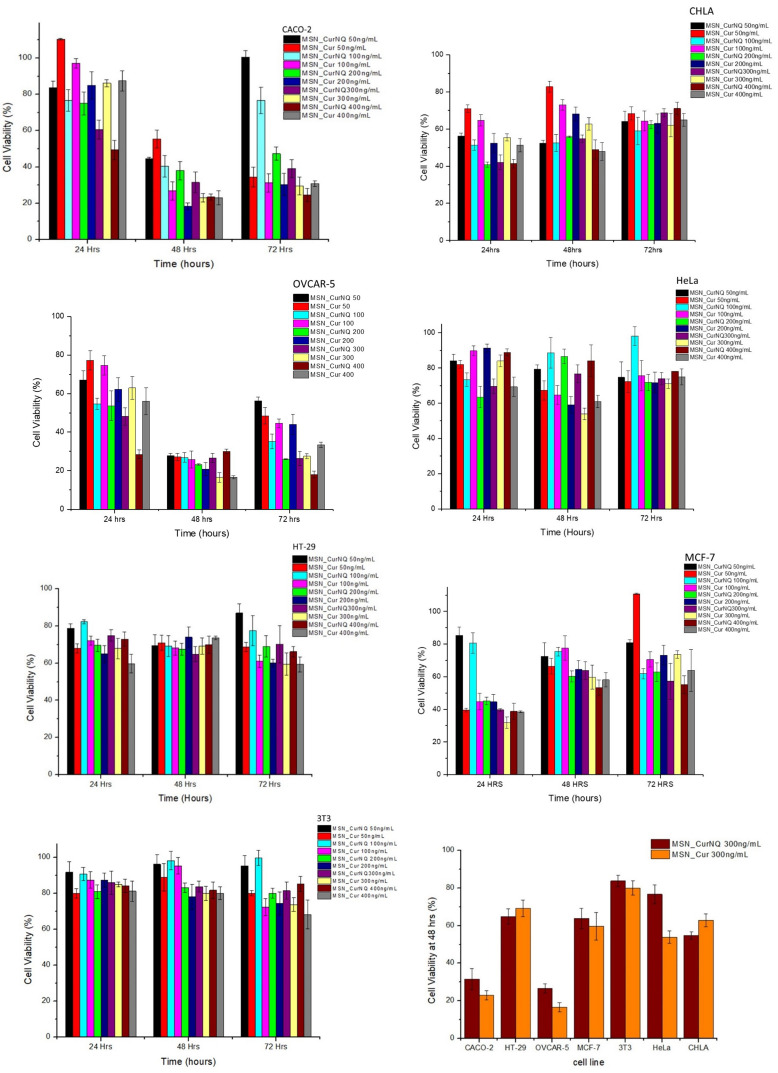
Cytotoxicity of MSN_CurNQ and MSN_Cur to CHLA, CACO-2, OVCAR-5, HeLa, HT-29, MCF-7, and 3T3 cell lines. MSN_CurNQ and MSN_Cur treatments were administered at a range of concentrations over a 72-h period and the reductions in cell viability were determined through an MTT assay. Statistically significant reductions in cell viability toward MSN_CurNQ treatment compared to the control cell line were observed for cell lines OVCAR-5, CACO-2, CHLA, HT-29, and MCF-7 cell lines.

As seen in Figures 8, the cytotoxicity of MSN_CurNQ and MSN_Cur depended greatly on the cancer cell type. Certain cancer cells such as CACO-2 and OVCAR-5 exhibited large reductions in cell viability, whereas other cancer cell lines such as HeLa experienced low overall toxicity in response to the loaded nanoparticles. Concentration administered and incubation time both affected overall cytotoxicity; however, cancer cell type was the largest determining factor.

**FIGURE 9:**
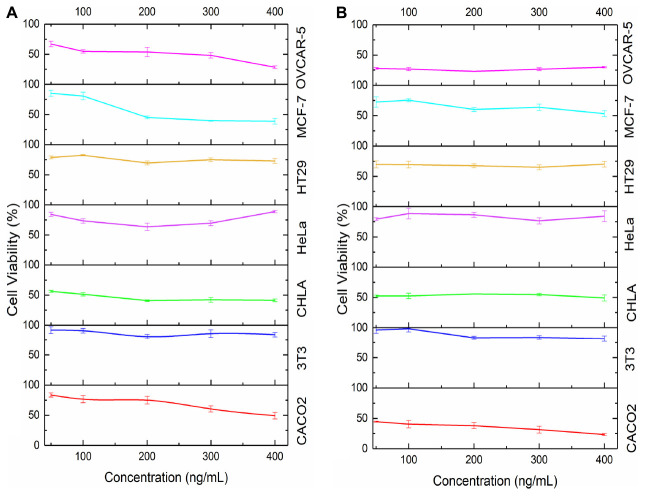
Cell viability in response to concentration of MSN_CurNQ after **(A)** 24 h and **(B)** 48 h in **six** cancer cell lines and in 1 healthy fibroblast cell line.

Cell viability was reduced to the greatest extent in the OVCAR-5 and CACO-2 cell lines. Cell viability dropped substantially after 48 h of incubation for both cell lines (Figure 9). This aligns with Figure 3 which demonstrated the prolonged release of CurNQ from the nanoparticles over a period of 96 h, thus the reduction in cell viability occurred once CurNQ was released. Release of CurNQ from the MSN increased drastically after 24 h (Figure 3), after which it would be able to exert its effects culminating in the decline in cell viability at 48 h. This sustained release is ideal for anti-cancer mechanisms, as it first allows for the nanoparticles to accumulate in the tumor prior to the complete release of the active compound. Additionally, this sustained release would allow for a longer detection window, in respect to the nanosystem’s proposed detection applications.

Although cell viability was reduced to the greatest extent at 48 h, at the 24-h incubation period, 400 ng/mL of MSN_CurNQ induced a reduction in cell viability to 28,5% in OVCAR-5 cells. This shows that this high concentration of CurNQ induced immediate cell death and this same response of immediate cell death occurred in the CACO-2 cell line. This suggests that enough CurNQ was released to induce cell death after 24 h on account of the high concentration administered. In the OVCAR-5 cell line, time was the most significant factor in the reduction of cell viability, as after 48 h, all treatment concentrations induced a reduction in cell viability to below 50%. However, cell recovery was observed after 72 h in cells receiving treatments of less than 200 ng/mL of MSN_CurNQ, whereas cell viability remained low after 72 h in cells that received 200 ng/mL or higher of MSN_CurNQ. This same pattern of cell recovery in response to low doses of the nanosystem was observed in CACO-2 cells, with the recovery being greater; but again, treatment with 200 ng/mL or greater induced sustained reductions in cell viability. Therefore, higher concentrations of treatment are required to induce lasting cell death.

MCF-7 and CHLA cell lines also demonstrated a reduction in cell viability but this reduction was slightly less compared to OVCAR-5 and CACO-2, yet cell viability was still reduced to below 50%, hence MSN_CurNQ shows promise as a broad chemotherapeutic agent that has the ability to act against a variety of cancer types. Interestingly, the MCF-7 and CHLA cell lines experienced their greatest reduction in cell viability after 24 h, differing from the response observed for OVCAR-5 and CACO-2 cell lines. While the release from MSN peaks after 96 h, there is not a large difference in release between 24 and 48 h on account of the sustained release from the nanoparticles. The MCF-7 and CHLA cell lines had an immediate response to treatment, with cell viability reducing to below 50% in cells receiving 200 ng/mL or higher of MSN_CurNQ. However, MCF-7 and CHLA cells recovered subsequent to their initial reduction in cell viability, and this also occurred in cells receiving the highest concentration of MSN_CurNQ; yet cells do not recover to full viability suggesting treatment was still effective in reducing the overall cell population. Although cell viability reduction was not as high as OVCAR-5 and CACO-2, the reduction in cell viability of MCF-7 and CHLA was statistically significantly different to the control cell line 3T3 (*p* = 0.000), indicating MSN_CurNQ treatment does result in a statistically significant reduction in cell viability to a variety of cancer types. Accordingly, it is recommended that in an *in vivo* setting, that a treatment regime is instigated, whereby a patient would receive several doses of the nanosystem, as to ensure malignant cell recovery does not occur.

There was a slight reduction in cell viability in HT-29 cells, cell viability was reduced to around 70%; and very low reductions in cell viability were observed for the HeLa cell line. This small reduction in cell viability precludes MSN_CurNQ’s chemotherapeutic application for these cancer types, as a greater reduction in cell viability are required for a system to be considered to have chemotherapeutic applications. On the contrary, MSN_CurNQ can still be applied as a detection and targeting system in these cancers. Owing to the multifaceted nature of MSN_CurNQ, its applications are not limited to a select few cancer types. Indeed, further treatment options would be required for cancer types that are not adequately treated by MSN_CurNQ; however, its application as a first line detection and targeting system is applicable.

Statistical analyses were performed to ascertain the effect of MSN_CurNQ and MSN_Cur on a variety of cancer cell types and this reduction was compared to the healthy fibroblast cell line 3T3. Statistical analyses were performed using Stata/IC 15.1 statistical software. The cell line was indeed a statistically significant factor for the reduction of cell viability (*p* = 0.0000). Administered concentration was also a statistically significant factor in cell viability reduction (*p* = 0.0000). OVCAR-5 cells were the most responsive to MSN_CurNQ treatment, and induced cell death was significantly different to each other cell line tested (HeLa, CHLA, MCF-7, HT-29, and 3T3 *p* = 0.000; CACO-2 *p* = 0.035). Importantly, there was a significant difference in the reduction in cell viability of the healthy cell line 3T3 to all other cancer cell lines investigated, barring HeLa cells (OVCAR-5, CACO-2, MCF-7, CHLA *p* = 0.000, HT-29 *p* = 0.004; HeLa *p* = 0.378). This means that MSN_CurNQ reduced cancer cell viability to a greater extent than it did to a healthy fibroblast cell line. The *p*-values of 0.000 and 0.004 indicate a high degree of statistically significance and verify the selective toxicity of MSN_CurNQ to cancer cells, meaning MSN_CurNQ has a higher potency toward cancerous cells than to healthy fibroblast cells. This selective toxicity further extends the cancer targeting capabilities of the nanosystem and further cements its novelty and applicability in the field of cancer theranostics.

The obtained results indicate a high concentration of MSN_CurNQ is required for effective and sustained reductions in cell viability. This high required dosage would not be a limiting factor to successful treatment on account of the observed cancer selective toxicity. Cell viability remained above 80% for the healthy fibroblast cell line 3T3, after treatment with 400 mg/mL of MSN_CurNQ, thereby indicating that MSN_CurNQ is non-toxic to these healthy fibroblast cells. This suggests that it would be safe to administer high concentrations of MSN_CurNQ to patients, as this should induce large reductions in cancer cell viability and a very low reduction to healthy cell viability.

Indeed, this research demonstrated that CurNQ requires to be loaded onto a nanoplatform to allow for all its theranostic capabilities to be realized. MSN_CurNQ thereby offers a three-pronged approach for cancer targeting namely its pH-responsive solubility, the EPR effect and its selective toxicity toward cancer cells. This makes MSN_CurNQ a highly advanced and novel nanosystem for cancer theranostics.

## Conclusion

The loading of CurNQ onto the MSN platform aimed to allow for enhanced delivery and enable *in vitro* and *in vivo* imaging and detection. To this end, MSN were synthesized and were impregnated with CurNQ to form the novel nanosystem MSN_CurNQ. CurNQ demonstrated pH responsive release from MSN, specific to the tumor microenvironment whereby after 96 h 31.5% of CurNQ was released at pH 7.4 compared to 57% release at pH 6.8, which thereby may allow for tumor targeting applications owing to pH responsivity. Moreover, MSN_CurNQ exhibited high intense, clear and distinctive innate fluorescence enabling detection and possible imaging applications. Moreover, MSN_CurNQ induced cytotoxicity culminating in a reduction to below 50% cell viability in OVCAR-5, CACO-2, CHLA, and MCF-7 cell lines thereby demonstrating its chemotherapeutic potential to a variety of cancer cell types. Furthermore, MSN_CurNQ treatment did not induce cytotoxicity to the healthy fibroblast cell line 3T3, demonstrating cancer selective toxicity, furthering the nanosystem’s novelty and potential applications. Clearly, the novel nanosystem MSN_CurNQ has potential applications in cancer theranostics.

## Data Availability Statement

The raw data supporting the conclusions of this article will be made available by the authors, without undue reservation.

## Author Contributions

PK, PP, and YC conceptualized the research. LF, TM, and PP contributed to methods and experimentation. LF wrote the first draft. PK and YC reviewed and revised the manuscript. All authors approved the final draft.

## Conflict of Interest

The authors declare that the research was conducted in the absence of any commercial or financial relationships that could be construed as a potential conflict of interest.
